# Breeding of yeast strains with intracellular amino acid accumulation for value-added alcoholic beverages

**DOI:** 10.1093/femsyr/foaf065

**Published:** 2025-10-29

**Authors:** Shota Isogai, Akira Nishimura, Hiroshi Takagi

**Affiliations:** Institute for Research Initiatives, Nara Institute of Science and Technology, 8916-5 Takayama-cho, Ikoma, Nara 630-0192, Japan; Department of Food and Agricultural Sciences, Faculty of Agriculture, Iwate University, 3-18-8, Ueda, Morioka, Iwate 020-8550, Japan; Institute for Research Initiatives, Nara Institute of Science and Technology, 8916-5 Takayama-cho, Ikoma, Nara 630-0192, Japan

**Keywords:** *Saccharomyces cerevisiae*, functional amino acids, alcoholic beverage, conventional mutagenesis, feedback inhibition

## Abstract

The yeast *Saccharomyces cerevisiae* converts amino acids into volatile compounds with fruity and floral aromas during fermentation. These amino acid-derived aroma compounds play a critical role in defining the taste and flavor of alcoholic beverages such as sake, beer, and wine. The productivity of amino acid-derived aroma compounds depends on the intracellular availability of their precursor amino acids. Therefore, breeding yeast strains that accumulate amino acids provides a practical approach to developing alcoholic beverages with more unique and attractive sensory characteristics. In this minireview, we describe the isolation of yeast strains that overproduce branched-chain amino acids and phenylalanine, obtained through conventional mutagenesis of industrial brewing yeasts. We also discuss the mechanisms responsible for the increased production of these amino acids in the mutant strains, including altered feedback regulation and transcriptional control of key enzymes involved in their biosynthesis. In addition, we briefly introduce a plasmid-free genome editing system that enables precise modification of metabolic pathways without the integration of foreign DNA, allowing the construction of strains that are not classified as genetically modified organisms. This method represents a promising tool that allows flexible and fine-tuned engineering of yeast metabolic pathways, including the development of strains with tailored aroma profiles.

## Teaser

This minireview summarizes our recent work on breeding amino acid-overproducing yeast strains by conventional mutagenesis for value-added alcoholic beverage production, along with elucidating the mechanisms responsible for amino acid overproduction in these mutants.

## Introduction

During the fermentation of alcoholic beverages, the yeast *Saccharomyces cerevisiae* not only converts glucose into ethanol but also produces various metabolites, such as higher alcohols, esters, and organic acids, that contribute to the taste and flavor of the final products. For instance, 2-phenylethanol exhibits a rose-like aroma, while 3-methylbutyl acetate and ethyl caproate emit a banana and an apple-like aroma and serve as major components of the *ginjo-ko* aroma, a distinctive aroma in Japanese sake. Thus, the development of new yeast strains that can enhance the diversity of flavor and aroma of alcoholic beverages is a promising approach to meet the demand for product differentiation. Although the modulation of metabolic pathways by introducing mutation(s) with foreign DNA is an effective strategy to construct desired yeast strains, at least in Japan, the public acceptance of food production using genetically modified organisms (GMOs) is still limited. Therefore, conventional mutagenesis with some chemicals or ultraviolet light seems to be the most practical and socially acceptable strategy to breed yeast strains for use in food and beverage production under the current societal constraints. For instance, aroma components in sake have been successfully modified through breeding approaches without genetic modifications (Negoro and Ishida 2022). Additionally, novel mutation-screening technologies, such as FIND-IT (Fast Identification of Nucleotide variants by droplet digital PCR) method have been developed to accelerate the selection of brewing yeast strains with desirable metabolic traits and altered flavor profiles (Stovicek et al. 2024). Alternatively, non-*Saccharomyces* yeast species such as *Hansenula, Kluyveromyces, Dekkera*, and *Lachancea* have been used in the production of wine, beer, and whisky to enhance and diversify their flavor profiles (Daute et al. 2024).

**Figure 1.**
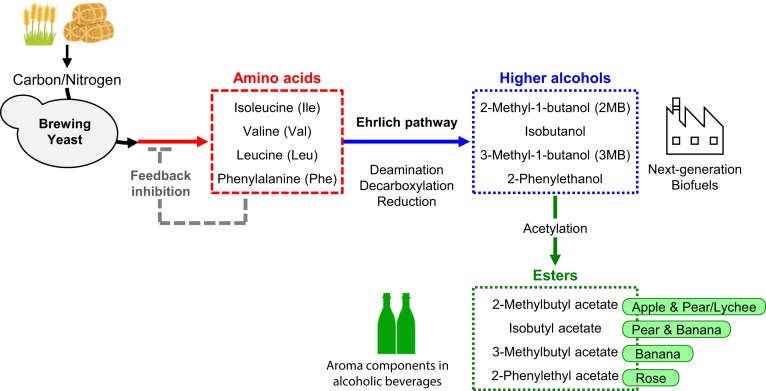
Metabolic pathway of aroma compounds derived from amino acids in *S. cerevisiae*. Branched-chain amino acids (isoleucine, valine, and leucine) and phenylalanine are converted to higher alcohols via deamination, decarboxylation, and reduction through the Ehrlich pathway. These higher alcohols are subsequently acetylated to form esters with fruity and floral aromas.

Some of the volatile compounds that contribute to the taste and flavor of alcoholic beverages are derived from amino acids via endogenous metabolic pathways, namely the Ehrlich pathway, in *S. cerevisiae* (Hazelwood et al. 2008). For example, isoleucine (Ile), valine (Val), and leucine (Leu), known as branched chain amino acids (BCAAs), along with phenylalanine (Phe) are catabolized via the Ehrlich pathway to yield 2-methyl-1-butanol (2 MB), isobutanol, 3-methyl-1-butanol (3 MB), and 2-phenylethanol, respectively. The Ehrlich pathway consists of three sequential enzymatic steps: deamination of amino acids to α-keto acids, followed by decarboxylation of α-keto acids to aldehydes, and finally reduction of aldehydes to higher alcohols (Fig. 1). These higher alcohols are further converted into acetate esters by alcohol acetyltransferases. Since amino acid-derived volatiles have characteristic fruity and floral aromas, the composition of these volatile compounds has a great impact on the flavor and taste of alcoholic beverages (Lilly et al. 2006, Pires et al. 2014). The production of these volatile compounds depends on the availability of their precursor amino acids in yeast cells. Therefore, yeast strains that accumulate these precursors are desired for production of unique and distinctive alcoholic beverages with enhanced flavor profiles. However, the overproduction of BCAAs and Phe is limited by metabolic regulatory mechanisms, such as feedback inhibition of biosynthetic enzymes and transcriptional activation of catabolic genes. To overcome this limitation without genetic engineering approaches, we isolated yeast mutants resistant to toxic amino acid analogues from randomly mutagenized cells. Due to their structural similarity, these analogues can be misincorporated into proteins instead of the corresponding amino acids, causing protein dysfunction, and subsequently leading to cell death. Overproduction of the corresponding amino acids can avoid incorrect incorporation of the toxic analogues, thereby alleviating growth inhibition. Thus, mutant strains resistant to the toxic amino acid analogues are expected to synthesize a large amount of corresponding amino acids. Furthermore, these analogue-resistant strains are advantageous for use in food and beverage production because the yeast cells do not contain any foreign genetic elements.

In this minireview, we describe our approach to isolating brewing yeast strains that overproduce BCAAs and Phe, used in the fermentation of sake, awamori, and beer, by conventional mutagenesis. We also discuss the mechanisms underlying increased amino acid productivity in the mutant strains. Moreover, we introduce a plasmid-free CRISPR/Cas9 system in *S. cerevisiae* to engineer metabolic pathways without introducing foreign DNA.

## Isoleucine

**Figure 2.**
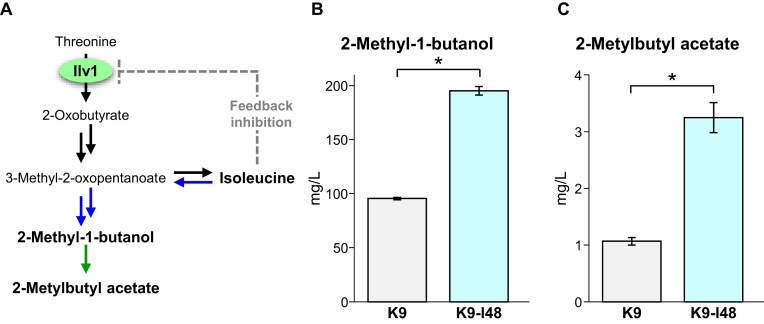
Isoleucine metabolic pathway (A) and concentrations of isoleucine-derived aroma compounds in sake (B, C). (A) Isoleucine metabolic pathway in *S. cerevisiae*. (B, C) Concentrations of 2-methyl-1-butanol (B) and 2-methylbutyl acetate (C) in sake making with strain K9-I48. The figure was redrawn based on data from Isogai et al. (2022). *P < .05.

In the fermentation process, Ile is catabolized via the Ehrlich pathway into 2 MB and subsequently converted to 2-methylbutyl acetate, which has an apple, pear, or lychee-like flavor. Ile is synthesized from threonine in five enzymatic steps (Binder et al. 2007, Amorim and Blanchard 2017). In yeast, the threonine deaminase Ilv1 catalyzes the first reaction in Ile biosynthesis, which is the deamination of threonine to produce 2-oxobutyrate. This deamination reaction is the rate-limiting step in Ile biosynthesis because the enzymatic activity of Ilv1 is subjected to allosteric inhibition by Ile, which prevents overproduction of Ile (Fig. 2A) (Holmberg and Litske 1988, Ernst and Downs 2018). First, we isolated mutants resistant to the Ile-toxic analogue *O*-methyl threonine (OMT) from the diploid sake yeast strain Kyokai no.9 (strain K9) by conventional mutagenesis. Among 100 OMT-resistant mutants, strain K9-I48 produced 2.9-fold more Ile than the parental strain. Given that Ilv1 catalyzes the rate-limiting step in Ile biosynthesis, we hypothesized that the elevated Ile production in K9-I48 might result from a mutation in the *ILV1* gene. Indeed, sequencing analysis of the *ILV1* gene of strain K9-I48 identified a novel mutation causing a His480Tyr substitution. The detailed analyses of the His480Tyr variant demonstrated that this substitution abolished the sensitivity to feedback inhibition by Ile. As a result, yeast expressing this variant produced more Ile and 2 MB than cells harboring the wild-type Ilv1. Furthermore, sake making with strain K9-I48 contained 2- to 3-fold more 2 MB and 2-methylbutyl acetate than sake making with the parent strain (Fig. 2B and C), indicating that this mutant strain contributes to the development of a new and distinctive sake flavor (Isogai et al. 2022).

## Valine

**Figure 3.**
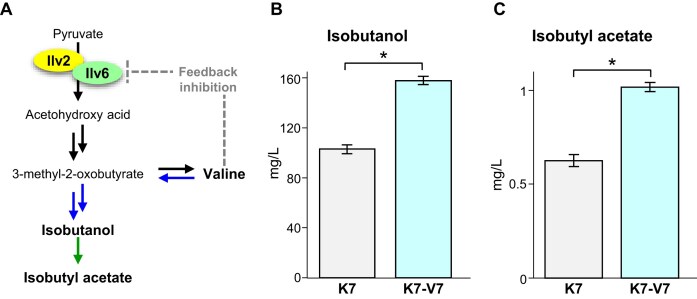
Valine metabolism pathway (A) and concentrations of valine-derived aroma compounds in sake (B, C). (A) Valine metabolic pathway in *S. cerevisiae*. (B, C) Concentrations of isobutanol (B) and isobutyl acetate (C) in sake making with strain K7-V7. The figure was redrawn based on data from Isogai et al. (2023). *P < .05.

Acetohydroxy acid synthase (AHAS) catalyzes the first reaction in Val biosynthesis, forming acetohydroxy acid by decarboxylative condensation of two molecules of pyruvate (McCourt and Duggleby 2006). AHAS of *S. cerevisiae* consists of two subunits: a catalytic subunit Ilv2 that catalyzes decarboxylation and a regulatory subunit Ilv6 that enhances the catalytic activity of Ilv2 (Pang and Duggleby 1999). Binding of the allosteric inhibitor Val to Ilv6 induces destabilization of the Ilv2-Ilv6 complex, thereby deactivating AHAS and preventing overproduction of Val in yeast cells (Lonhienne et al. 2020). Moreover, intracellular Val is converted to isobutanol via the Ehrlich pathway and then to isobutyl acetate (Fig. 3A). Since isobutyl acetate imparts a pear- and banana-like aroma, Val-derived aroma compounds have a significant impact on the flavor of alcoholic beverages. To enhance Val-derived flavor compounds in sake, we randomly mutagenized the diploid sake yeast Kyokai no.7 (strain K7) and obtained a mutant strain, K7-V7, that produced 1.7-fold more Val than the parental strain. The nucleotide sequence of the *ILV6* gene revealed a heterozygous mutation, resulting in an amino acid substitution of alanine with threonine at position 31 (Ala31Thr). Enzymatic analysis indicated that the Ala31Thr substitution in Ilv6 decreased sensitivity to feedback inhibition by Val, leading to Val accumulation and increased isobutanol production in the laboratory yeast cells expressing this variant. Moreover, sake making with strain K7-V7 contained 1.5-fold more isobutanol and isobutyl acetate than sake making with the parental strain (Fig. 3B and C), contributing to the making of differentiated sake with a fruity aroma originated from Val-derived volatiles (Isogai et al. 2023).

## Leucine

**Figure 4.**
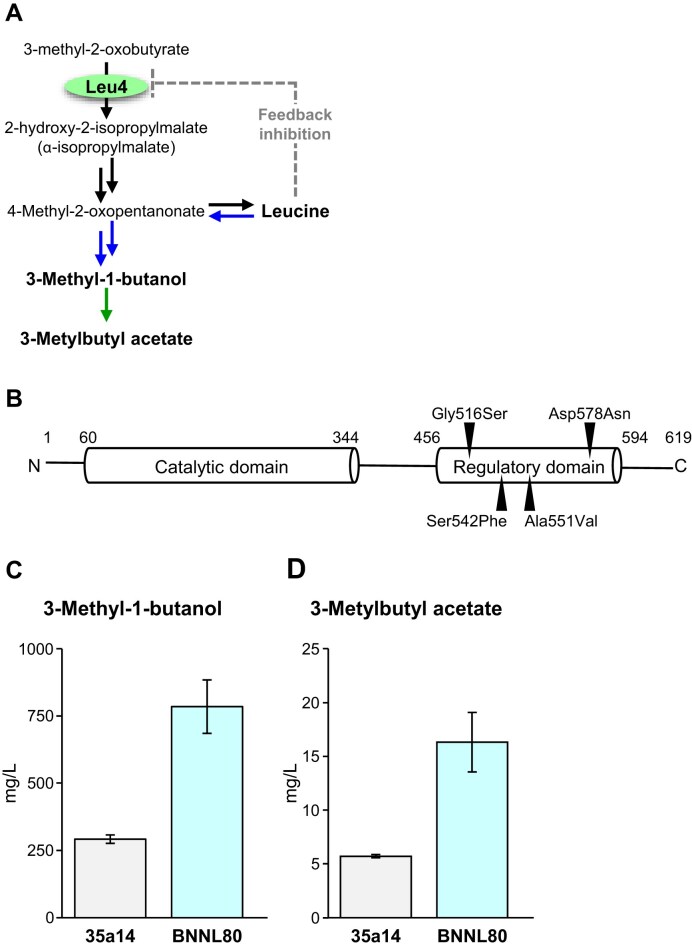
Leucine metabolism pathway (A), domain organization of Leu4 (B), and concentrations of leucine-derived aroma compounds in awamori (C, D). (A) Leucine metabolic pathway in *S. cerevisiae*. (B) Domain organization of Leu4. Black triangles indicate amino acid substitutions identified in leucine-accumulating mutant strains. (C, D) Concentrations of 3-methyl-1-butanol (C) and 3-methylbutyl acetate (D) in awamori brewed using a leucine-accumulating mutant (BNNL80) derived from a yeast isolate originating from banana stems (35a14). The figure was redrawn based on data from Tsukahara et al. (2023).

During fermentation, Leu is catabolized via the Ehrlich pathway to 3 MB, which is further converted into 3-methylbutyl acetate, a volatile compound known for its strong fruity aroma. These compounds are recognized as important contributors to the fruity aroma of awamori (Taira et al. 2012), a traditional distilled alcoholic beverage produced in Okinawa, Japan. Therefore, we isolated Leu-accumulating mutants from three yeast strains: one traditionally used for awamori brewing, and two wild-type strains obtained from hibiscus flowers and banana stems. All Leu-accumulating mutants had mutations in the *LEU4* gene, which encodes α-isopropylmalate synthase (IPMS). IPMS catalyzes the condensation of 3-methyl-2-oxobutyrate and acetyl-coenzyme A to form α-isopropylmalate (2-hydroxy-2-isopropylmalate) (Fig. 4A) (Baichwal et al. 1983). Since feedback inhibition of IPMS by Leu suppresses Leu accumulation in yeast cells, we analyzed the nucleotide sequence of the *LEU4* gene to identify mutations that cause Leu overproduction. Amino acid substitutions in the Leu4 protein, such as Ser542Phe, Ala551Val (Takagi et al. 2015), Gly516Ser (Abe et al. 2019), and Asp578Asn (Tsukahara et al. 2023), were found to contribute to Leu overproduction in yeast, leading to increased production of Leu-derived aromas in awamori (Fig. 4B–D).

## Phenylalanine

**Figure 5.**
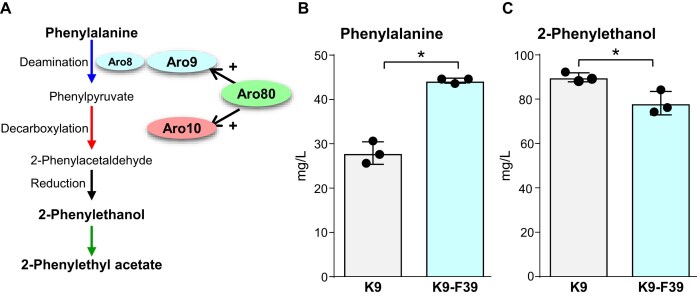
Phenylalanine metabolism pathway (A) and concentrations of phenylalanine and its derivative in sake (B, C). (A) Phenylalanine metabolic pathway in *S. cerevisiae*. (B, C) Concentrations of phenylalanine (B) and 2-phenylethanol (C) in sake making with strain K9-F39. The figure was redrawn based on data from Nishimura et al. (2022). *P < .05.

In *S. cerevisiae* cells, Phe is metabolized through the Ehrlich pathway to produce 2-phenylethanol and its acetate ester, 2-phenylethyl acetate. These compounds are characterized by a rose-like aroma and are the main components of the floral notes in fermented beverages such as sake and beer. In the Ehrlich pathway, the transcription of the *ARO9* and the *ARO10* genes, which encode deaminase and decarboxylase, respectively (Iraqui et al. 1998), is activated by the transcription factor Aro80 in response to intracellular aromatic amino acids (Fig. 5A) (Iraqui et al. 1999). We isolated a Phe-accumulating strain, K9-F39, from mutants resistant to the toxic Phe analogue, *p*-fluorophenylalanine, which were derived from the industrial sake yeast strain K9. Whole-genome sequencing analysis of strain K9-F39 revealed a novel mutation in the *ARO80* gene encoding the His309Gln variant. Functional analysis demonstrated that this variant retained activity to bind to the *ARO9* and *ARO10* promoters, but was unable to activate their transcription. As a result, Phe degradation was suppressed, leading to intracellular Phe accumulation in strain K9-F39. Furthermore, Phe content in sake making with strain K9-F39 substantially increased compared to that of the parental strain, whereas the level of 2-phenylethanol was slightly decreased (Fig. 5B and C). These results suggest that regulating the expression of the *ARO9* and *ARO10* genes via the Aro80 variant allows control of Phe and its derived aroma compounds in sake, providing a strategy for making differentiated sake (Nishimura et al. 2022).

Furthermore, we recently isolated a Phe-accumulating beer yeast using a similar breeding strategy. Beer brewed with this mutant strain showed significant increases in 2-phenylethanol and 2-phenylethyl acetate. This led to the commercialization of a highly aromatic craft beer, which was released by a local brewery in Nara in the fall of 2022.

## Development of a non-GMO genome editing system for yeast breeding

Although conventional mutagenesis is still widely used in microbial breeding, it carries an inherent risk of introducing unintended or unfavorable mutations that impair beneficial traits, such as ethanol production and stress tolerance. It is desirable to introduce only beneficial mutations that enhance the function of key biosynthetic enzymes, such as those described in this minireview, because this approach can prevent unintended mutations that compromise fermentation ability. Genome editing techniques such as CRISPR/Cas9 are powerful and suitable tools for such site-directed mutagenesis. However, these techniques typically require the use of foreign DNA (e.g. plasmids), which currently limits their application in food production in Japan due to a lack of social acceptance and regulatory clarity. To enable targeted genome editing without introducing foreign DNA, we developed a plasmid-free genome editing system that employs cell-penetrating peptides to directly deliver the Cas9-guide RNA complex into the cytoplasm of laboratory yeast strains (Nishimura et al. 2023). Nevertheless, several challenges remain for the practical use of genome-edited yeast strains in alcoholic-beverage production. First, further improvement of editing efficiency and establishment of social consensus are required. In addition, although genome-editing technologies have been applied to the breeding of crops and microbes for food and beverage production in Japan since 2019 (Tsuda et al. 2019), relevant legal frameworks related to safety assessment and labeling are still under discussion among the relevant ministries. Moreover, with respect to intellectual property, the use of the CRISPR/Cas9 system for commercial applications may require licenses from overseas patent holders. To avoid potential intellectual-property restrictions, domestically developed genome-editing technologies that do not infringe on the Cas9 patent family, such as the Target-AID/nCas9 base-editing system (Nishida et al. 2016) and the CRISPR Type I-D (TiD) system (Osakabe et al. 2020), could be considered, given that these Japan-origin platforms. Combining our plasmid-free system with these genome-editing technologies is expected to enable efficient breeding of non-GMO yeast strains with desired traits including improved flavor production.

## Conclusions and outlook

In this minireview, we summarized our approach to enhancing the aromatic profiles of alcoholic beverages through yeast breeding focused on amino acid metabolism. By isolating mutant strains resistant to toxic amino acid analogues by conventional mutagenesis, we developed yeast strains that accumulate amino acids as precursors of flavor components in alcoholic beverages. This strategy, which combines amino acid analogues with mutagenesis, is also applicable to value-added production in a wide range of fermented foods beyond alcoholic beverages. Higher alcohols and their esters derived from amino acids are not only used as flavor and fragrance compounds in foods and cosmetics but are also attracting attention as next-generation biofuels. Therefore, it is expected that the basic knowledge gained from the analysis of amino acid-accumulating yeast mutants will contribute to improving the fermentation-based production of biofuels and cosmetic ingredients.

In this minireview, we have focused on amino acids as precursors of aroma compounds, whereas intracellular free amino acids also possess various functional properties, such as cytoprotective effects. We have recently named this breeding approach “amino acid functional engineering,” which aims to enhance the production of such functional amino acids and thereby improve the productivity of valuable compounds and the performance of various organisms, including microorganisms and plants (Takagi 2019). Our current research aims to establish this strategy and contribute to future biotechnological applications.

## References

[bib1] Abe T, Toyokawa Y, Sugimoto Y et al. Characterization of a new *Saccharomyces cerevisiae* isolated from hibiscus flower and its mutant with l-leucine accumulation for awamori brewing. Front Genet. 2019;10:490. 10.3389/fgene.2019.00490. 31231421 PMC6558412

[bib2] Amorim FTM, Blanchard JS. Bacterial branched-chain amino acid biosynthesis: structures, mechanisms, and drugability. Biochemistry. 2017;56:5849–65. 10.1021/acs.biochem.7b00849. 28977745 PMC5839172

[bib3] Baichwal VR, Cunningham TS, Gatzek PR et al. Leucine biosynthesis in yeast. Curr Genet. 1983;7:369–77. 10.1007/BF00445877. 24173418

[bib4] Binder S, Knill T, Schuster J. Branched-chain amino acid metabolism in higher plants. Physiol Plant. 2007;129:68–78. 10.1111/j.1399-3054.2006.00800.x.

[bib5] Daute M, Jack F, Walker G. The potential for Scotch Malt Whisky flavour diversification by yeast. FEMS Yeast Res. 2024;24:foae017. 10.1093/femsyr/foae017. 38684485 PMC11095643

[bib6] Ernst DC, Downs DM. Mmf1p couples amino acid metabolism to mitochondrial DNA maintenance in *Saccharomyces cerevisiae*. mBio. 2018;9:e00084–18. 10.1128/mBio.00084-18. 29487232 PMC5829821

[bib7] Hazelwood LA, Daran J-M, van Maris AJA et al. The Ehrlich pathway for fusel alcohol production: a century of research on *Saccharomyces cerevisiae* metabolism. Appl Environ Microb. 2008;74:2259–66. 10.1128/AEM.02625-07. PMC2293160 18281432

[bib8] Holmberg S, Litske PJG. Regulation of isoleucine-valine biosynthesis in *Saccharomyces cerevisiae*. Curr Genet. 1988;13:207–17. 10.1007/BF00387766. 3289762

[bib9] Iraqui I, Vissers S, André B et al. Transcriptional induction by aromatic amino acids in *Saccharomyces cerevisiae*. Mol Cell Biol. 1999;19:3360–71. 10.1128/MCB.19.5.3360. 10207060 PMC84129

[bib10] Iraqui I, Vissers S, Cartiaux M et al. Characterisation of *Saccharomyces cerevisiae ARO8* and *ARO9* genes encoding aromatic aminotransferases I and II reveals a new aminotransferase subfamily. Mol Gen Genet MGG. 1998;257:238–48. 10.1007/s004380050644. 9491083

[bib11] Isogai S, Nishimura A, Kotaka A et al. High-level production of isoleucine and fusel alcohol by expression of the feedback inhibition-insensitive threonine deaminase in *Saccharomyces cerevisia*e. Appl Environ Microb. 2022;88:e02130–21. 10.1128/aem.02130-21. PMC8904041 35020456

[bib12] Isogai S, Nishimura A, Murakami N et al. Improvement of valine and isobutanol production in sake yeast by Ala31Thr substitution in the regulatory subunit of acetohydroxy acid synthase. FEMS Yeast Res. 2023;23:foad012. 10.1093/femsyr/foad012. 36812944

[bib13] Lilly M, Bauer FF, Styger G et al. The effect of increased branched-chain amino acid transaminase activity in yeast on the production of higher alcohols and on the flavour profiles of wine and distillates. FEMS Yeast Res. 2006;6:726–43. 10.1111/j.1567-1364.2006.00057.x. 16879424

[bib14] Lonhienne T, Low YS, Garcia MD et al. Structures of fungal and plant acetohydroxyacid synthases. Nature. 2020;586:317–21. 10.1038/s41586-020-2514-3. 32640464

[bib15] McCourt JA, Duggleby RG. Acetohydroxyacid synthase and its role in the biosynthetic pathway for branched-chain amino acids. Amino Acids. 2006;31:173–210. 10.1007/s00726-005-0297-3. 16699828

[bib16] Negoro H, Ishida H. Development of sake yeast breeding and analysis of genes related to its various phenotypes. FEMS Yeast Res. 2022;22:foac057. 10.1093/femsyr/foac057. 36370450

[bib17] Nishida K, Arazoe T, Yachie N et al. Targeted nucleotide editing using hybrid prokaryotic and vertebrate adaptive immune systems. Science. 2016;353:aaf8729. 10.1126/science.aaf8729. 27492474

[bib18] Nishimura A, Isogai S, Murakami N et al. Isolation and analysis of a sake yeast mutant with phenylalanine accumulation. J Ind Microbiol Biotechnol. 2022;49:kuab085. 10.1093/jimb/kuab085. 34788829 PMC9142190

[bib19] Nishimura A, Tanahashi R, Oi T et al. Plasmid-free CRISPR/Cas9 genome editing in *Saccharomyces cerevisiae*. Biosci Biotechnol Biochem. 2023;87:458–62. 10.1093/bbb/zbad008. 36694939

[bib20] Osakabe K, Wada N, Miyaji T et al. Genome editing in plants using CRISPR type I-D nuclease. Commun Biol. 2020;3:648. 10.1038/s42003-020-01366-6. 33159140 PMC7648086

[bib21] Pang SS, Duggleby RG. Expression, purification, characterization, and reconstitution of the large and small subunits of yeast acetohydroxyacid synthase. Biochemistry. 1999;38:5222–31. 10.1021/bi983013m. 10213630

[bib22] Pires EJ, Teixeira JA, Brányik T et al. Yeast: the soul of beer’s aroma—A review of flavour-active esters and higher alcohols produced by the brewing yeast. Appl Microbiol Biotechnol. 2014;98:1937–49. 10.1007/s00253-013-5470-0. 24384752

[bib23] Stovicek V, Lengeler KB, Wendt T et al. Modifying flavor profiles of *Saccharomyces* spp. for industrial brewing using FIND-IT, a non-GMO approach for metabolic engineering of yeast. N Biotechnol. 2024;82:92–106. 10.1016/j.nbt.2024.05.006. 38788897

[bib24] Taira J, Tsuchiya A, Furudate H. Initial volatile aroma profiles of young and aged awamori shochu determined by GC/MS/Pulsed FPD. Food Sci Technol Res. 2012;18:177–81. 10.3136/fstr.18.177.

[bib25] Takagi H, Hashida K, Watanabe D et al. Isolation and characterization of awamori yeast mutants with l-leucine accumulation that overproduce isoamyl alcohol. J Biosci Bioeng. 2015;119:140–7. 10.1016/j.jbiosc.2014.06.020. 25060730

[bib26] Takagi H. Metabolic regulatory mechanisms and physiological roles of functional amino acids and their applications in yeast. Biosci Biotechnol Biochem. 2019;83:1449–62. 10.1080/09168451.2019.1576500. 30712454

[bib27] Tsuda M, Watanabe KN, Ohsawa R. Regulatory status of genome-edited organisms under the Japanese Cartagena Act. Front Bioeng Biotechnol. 2019;7:387. 10.3389/fbioe.2019.00387. 31867318 PMC6908812

[bib28] Tsukahara M, Isogai S, Azuma H et al. Characterization of a new *Saccharomyces cerevisiae* isolated from banana stems and its mutant with l-leucine accumulation for awamori brewing. Biosci Biotechnol Biochem. 2023;87:240–4. 10.1093/bbb/zbac185. 36396349

